# County-Level Food Insecurity and Hepatocellular Carcinoma Risk: A Cross-Sectional Analysis

**DOI:** 10.3390/ijerph22010120

**Published:** 2025-01-18

**Authors:** Rebecca D. Kehm, Chrystelle L. Vilfranc, Jasmine A. McDonald, Hui-Chen Wu

**Affiliations:** 1 Department of Epidemiology, Mailman School of Public Health of Columbia University, New York, NY 10032, USA; rk2967@cumc.columbia.edu (R.D.K.); jam2319@cumc.columbia.edu (J.A.M.); 2 Herbert Irving Comprehensive Cancer Center, Columbia University Medical Center, New York, NY 10032, USA; cv2529@cumc.columbia.edu; 3 Department of Environmental Health Sciences, Mailman School of Public Health of Columbia University, New York, NY 10032, USA

**Keywords:** food insecurity, hepatocellular carcinoma, social determinants of health, health disparities, Surveillance Epidemiology and End Results Program

## Abstract

Food insecurity (FI) is associated with several known hepatocellular carcinoma (HCC) risk factors, but few studies have directly examined FI in association with HCC risk. We aimed to investigate whether county-level FI is associated with HCC risk. We used data from 21 registries in the Surveillance Epidemiology and End Results database to obtain county-level counts of HCC cases from 2018 to 2021. We obtained the county-level FI rates for 2018–2021 from Feeding America’s Map the Meal Gap. We used multi-level Poisson regression models with robust standard errors to calculate incidence rate ratios (IRRs) and 95% confidence intervals (CIs). Overall, a one-standard-deviation (SD) increase in county-level FI was associated with an 8% increase in HCC risk in the fully adjusted model (IRR = 1.08, 95% CI = 1.06, 1.10). When stratified by age at diagnosis, a one-SD increase in county-level FI was associated with a 2% higher risk of HCC in the ≥65 age group (IRR = 1.02, 95% CI = 1.00, 1.05) and a 15% higher risk in the <65 age group (IRR = 1.15, 95% CI = 1.11, 1.19; interaction *p*-value < 0.001). If confirmed in other studies, these findings support the need for interventions and policies addressing FI in populations at increased risk for HCC.

## 1. Introduction

Hepatocellular carcinoma (HCC), the most common form of primary liver cancer, is the sixth most common cancer and the third leading cause of cancer-related deaths worldwide [1,2]. HCC is often diagnosed at a late stage, resulting in limited treatment options [3]; 50% of HCC patients die within 10 months [4], and nearly 80% die within five years [5]. However, survival rates have been improving over time, partly due to an increase in early-stage HCC cases detected through regular surveillance efforts [6]. In the U. S., HCC incidence rates have tripled over the past 20 years [7,8] and continue to rise [9,10]. Several well-known risk factors for HCC include hepatitis B virus (HBV), hepatitis C virus, liver fibrosis, alcohol use, and obesity [11,12,13]. Yet, HCC etiology has recently shifted from a higher proportion of viral causes to a higher proportion of non-viral causes [12,14]. Further, up to 40% of HCC cases are unexplained by known risk factors [14,15]. Additional research is thus needed to identify novel risk factors contributing to the increase in HCC incidence over time.

There are significant disparities in HCC incidence and survival, which suggests that social determinants of health play a role in the disease. Historically, Asian and Pacific Islanders in the U.S. had the highest rates of HCC, primarily due to the high prevalence of HBV infection among immigrants from HBV-endemic regions [16]. However, HCC rates in this group are now declining [17], which is attributed to lower HBV infection rates among U.S.-born generations, the success of HBV vaccination programs, and advancements in antiviral therapy for HBV [16]. In contrast, HCC incidence rates are increasing among other racial and ethnic groups, including non-Hispanic Blacks and Hispanics [18,19], who also experience shorter median survival times after HCC diagnosis [20,21,22,23]. Additionally, lower socioeconomic status has been associated with a higher risk of HCC, limited access to treatment, and poorer outcomes after diagnosis [24,25,26,27]. For example, a recent study found that 48% of the difference in HCC incidence rates between non-Hispanic Black neighborhoods and predominantly non-Hispanic White neighborhoods was attributed to neighborhood deprivation [28]. Similarly, 15% of the disparity between Hispanic neighborhoods and non-Hispanic White neighborhoods was due to neighborhood deprivation [28]. The mechanisms through which lower socioeconomic status increases HCC risk have not been fully elucidated. Possible factors include delayed access to regular HCC surveillance and medical care for treating underlying health conditions such as cirrhosis [29]. Financial constraints are one of the most common patient-reported barriers associated with HCC surveillance [30]. The link may also be due to how social factors influence known and emerging HCC risk factors, such as alcohol consumption, diet quality, and obesity [31,32,33]. More research is needed to identify modifiable factors contributing to these disparities to inform interventions aimed at reducing inequities in HCC.

We hypothesize that food insecurity (FI) may be a social determinant of health contributing to disparities in HCC. FI is defined as having limited or uncertain access to enough safe, nutritious, and acceptable food due to economic and social challenges at the household level [34]. In high-income countries like the U.S., inequality is the main cause of FI [35]. FI is considered a downstream result of broader structural determinants of health, and the risk for FI increases as financial hardship increases. For example, a study found that chronic liver disease patients who were unable to pay medical bills had nearly six times higher odds of FI compared to patients without financial hardship from medical bills [36]. FI is associated with several established and emerging risk factors for HCC, including poor diet quality [37], diabetes [38,39,40], heavy alcohol use [41,42], obesity [43], and metabolic syndrome [44,45]. Additionally, some evidence suggests that FI is common among individuals with metabolic dysfunction-associated steatotic disease (MASLD) [46,47,48,49,50,51], formerly known as non-alcohol fatty liver disease [52], a recognized risk factor for HCC [53]. A recent study using data from the 2017–2018 National Health and Nutrition Examination Survey (NHANES) found that FI was associated with a 42% increased odds of MASLD (odds ratio = 1.42, 95% CI = 1.12–1.78) and a 40% increased odds of hepatic fibrosis (odds ratio = 1.40, 95% CI = 1.04–1.88) [50]. Another longitudinal analysis of NHANES data found that FI was independently associated with higher all-cause mortality among individuals with chronic liver disease, even after controlling for other socioeconomic factors such as poverty, income, and education level [54]. Despite these findings, few studies have examined whether FI is directly associated with increased HCC risk. We addressed this research gap by conducting a cross-sectional analysis of population-based data to examine whether county-level FI is associated with county-level HCC incidence rates in the United States.

## 2. Methods

### 2.1. Study Population

We used data from the Surveillance, Epidemiology, and End Results (SEER) database to obtain county-level counts of HCC cases diagnosed from 2018 to 2021 across 21 registries, including: Atlanta (metropolitan), Connecticut, Greater California, Greater Georgia, Hawaii, Iowa, Idaho, Illinois, Kentucky, Los Angeles, Louisiana, Massachusetts, New Jersey, New Mexico, New York, Rural Georgia, San Franscico-Oakland, San Jose-Monterey, Seattle (Puget Sound), Texas, and Utah. These registries cover approximately 48% of the U.S. population, encompassing a total of 1085 counties [55]. The 2018–2021 timeframe was selected because it aligns with the most recent period for which consistent county-level FI measures are available (see below for more details). Ethical review and informed consent were not required for this study because only publicly available county-level data were used.

### 2.2. Hepatocellular Carcinoma

We identified HCC cases in the SEER database using the International Classification of Diseases for Oncology, Third Edition (ICD-O-3). We included cases with a topography code of C22.0 for primary liver cancer and ICD-O-3 histology codes 8170–8175. We excluded cases diagnosed solely through clinical evaluation (without microscopic confirmation) or identified only through autopsy or death certificate records. We further limited our analysis to cases recorded as the first primary cancer.

Within each county, we stratified HCC case counts by age at diagnosis (<65 years and ≥65 years), sex (female and male), race, and ethnicity (Hispanic, non-Hispanic Asian or Pacific Islander, non-Hispanic Black, and non-Hispanic White). We excluded cases diagnosed before the age of 20 years along with cases that identified as American Indian or Alaska Native due to small case counts. Additionally, we stratified case counts by stage at diagnosis, categorized as localized, regional, and distant, using the combined summary stage (2004+) variable in the SEER database [56].

### 2.3. Food Insecurity

We obtained county-level FI rates for 2018–2021 from Feeding America’s Mapping the Meal Gap reports (http://map.feedingamerica.org; accessed on 20 August 2023. additional details provided elsewhere [57]). Feeding America, a national hunger relief organization operating the largest network of food banks and pantries in the United States, conducts an annual study measuring FI levels for every county in the United States [57].

To assess FI, Feeding America uses data from the Core Food Security Module (CFSM) of the Current Population Survey (CPS) [57]. The Census Bureau administers the CPS for the Bureau of Labor Statistics to gather nationally representative data on employment, income, and poverty [58]. Each December, 50,000 households receive a version of the CPS that includes the CFSM [59]. Households with children answer 18 F1-related questions, while those without children answer 10 FI-related questions. Sample questions include: “I worried whether our food would run out before we got money to buy more.”; “Did you or the other adults in your household ever cut the size of your meals or skip meals because there wasn’t enough money for food?”; and “Were you ever hungry but did not eat because you couldn’t afford enough food?” [59]. The full list of questions in the CFSM is available elsewhere [59]. Households responding “yes” to three or more CFSM questions are classified as FI [59].

Feeding America estimates FI at the county-level using a two-step process [57,60]. First, they use Equation (1) to estimate state-level FI rates:

FI_st_ = α + β_UN_UN_st_ + β_POV_POV_st_ + β_MI_MI_st_ + β_HISP_HISP_st_ + β_BLACK_BLACK_st_ + β_OWN_OWN_st_ + β_DSBL_DSBL_st_ + µ_t_ + υ_s_ + ε_st_ (1)

In Equation (1), s represents a state and t represents a year. The variables are defined as follows: UN: unemployment rate, POV: poverty rate for non-undergraduate students, MI: median income, HISP: percentage of the population that is Hispanic, BLACK: percentage of the population that is Black or African American, OWN: percentage of individuals who are homeowners, DSBL: percentage of individuals reporting a disability [57]. These variables were selected based on existing research and data availability [57]. The model also included year (µ_t_) and state (υ_s_) fixed effects to account for unobserved, state- and year-specific factors that might influence FI. State population weights were used in the estimation [57,60].

Next, Feeding America applies the estimated coefficients from Equation (1) along with county-level data for these same variables to calculate FI rates at the county-level, as shown in Equation (2) [57]:

(2) $${\mathrm{FI}}_{c}=\hat{\alpha }+\hat{\beta_{UN}}{\mathrm{UN}}_{c}+\hat{\beta_{POV}}{\mathrm{POV}}_{c}+\hat{\beta_{MI}}{\mathrm{MI}}_{c}+\hat{\beta_{HISP}}{\mathrm{HISP}}_{c}+\hat{\beta_{BLACK}}{\mathrm{BLACK}}_{c}+\hat{\beta_{OWN}}{\mathrm{OWN}}_{c}+\hat{\beta_{DSBL}}{\mathrm{DSBL}}_{c}+\hat{\mu_{YEAR}}+\hat{\nu_{s}}$$

In Equation (2), c represents a county. The variables POV_c_, MI_c_, HISP_c_, BLACK_c_, OWN_c_, and DISBL_c_ are derived from 5-year estimates from the American Community Survey, while UN_c_ is based on 1-year averages from the Bureau of Labor Statistics [49]. Using Equation (2), Feeding America calculates FI rates for each county [57,60].

For this analysis, we obtained county-level annual FI rates from Feeding America for 2018-2021. We then calculated the average FI rate for each county over this period.

### 2.4. Covariates

We obtained additional county-level data on established HCC risk factors for 2018–2021, including rates of binge drinking (defined as consuming 4 or more drinks for women and 5 or more drinks for men on a single occasion [61]), current cigarette smoking, and obesity (body mass index ≥ 30 kg/m^2^). Individual-level data on these lifestyle factors were collected by the Behavior Risk Factor Surveillance System (BRFSS) [61], and county-level estimates were generated by the PLACES Project [62]. For each covariate, we calculated the average rate over the 2018–2021 period.

### 2.5. Statistical Analysis

To evaluate the association between county-level FI and HCC risk, we used Poisson multivariable regression models with robust variance estimation to calculate incidence rate ratios (IRRs) and 95% confidence intervals (CIs). County population size was used as the offset term. We used Poisson regression for this analysis because it is well-suited for modeling count data, such as the number of HCC cases, while accounting for differing population sizes across counties through the use of an offset term. We employed robust variance estimation to address potential violations of the model’s assumption that the mean and variance of the outcome are equal. Additionally, we included a random effect to account for potential state-level clustering, such as the influence of state policies on HCC risk. We modeled FI as both a continuous variable, standardized to a mean of zero and standard deviation of one, and categorized into tertiles based on the study sample distribution. To test for a linear trend across FI tertiles, we fitted a model treating the tertile variable as continuous. We included potential confounders as covariates in the model. We analyzed two models: Model 1 was adjusted for age at diagnosis, sex, race, and ethnicity; Model 2 included additional adjustments for county-level rates of binge drinking, smoking, and obesity. Both models included 17,204 observations, representing the total number of county-level estimates stratified by age group, sex, race, and ethnicity. To examine effect modification by sex, age at diagnosis, race, and ethnicity, we added cross-product terms to the models. We also analyzed models stratified by stage at diagnosis (localized, regional, and distant). All statistical tests were two-sided, and *p-*values below 0.05 were considered statistically significant. All analyses were performed using Stata 15.1 (College Station, TX, USA).

## 3. Results

**Figure 1:**
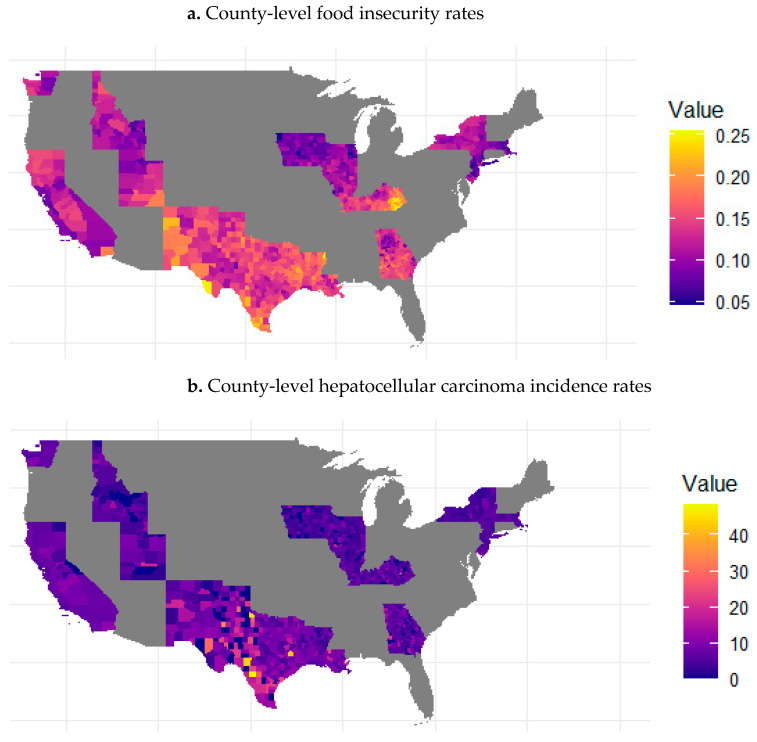
(**a**) County-level food insecurity rates and (**b**) county-level hepatocellular carcinoma incidence rates for the timeframe of 2018–2021 for each county within Surveillance, Epidemiology, and End Results catchment areas in the contiguous United States. The values in the heatmaps reflect rates per 100,000, with the lowest rates illustrated in dark purple and the highest rates illustrated in yellow.

There were 38,679 cases of HCC in the study sample diagnosed from 2018 to 2021. A total of 76 percent of cases were male, 48% of cases were non-Hispanic White, and 55% were aged 65 years and older at diagnosis (Table 1). The average FI rate was 12.9% (standard deviation (SD) = 3.6%) among counties included in the analysis. Figure 1 displays the geographical distribution of county-level FI rates and HCC incidence rates per 100,000 people across counties within the SEER catchment areas in the contiguous United States (excluding Hawaii).

Overall, a 1-SD increase in county-level FI was associated with a 13% higher risk of HCC, adjusting for the patient characteristics of sex, age at diagnosis, race, and ethnicity. (Table 2, Model 1: IRR = 1.13, 95% CI = 1.08, 1.18). This association changed to an 8% higher risk after further adjustment for county-level HCC risk factors, including binge drinking, smoking, and obesity rates (Model 2: IRR = 1.08, 95% CI = 1.06, 1.10). When county-level FI was categorized into tertiles, being in the highest versus lowest FI tertile was associated with a 28% higher risk of HCC in the minimally adjusted model (Model 1: IRR = 1.28, 95% CI = 1.10, 1.49; *p*-trend = 0.01), and a 12% higher risk in the fully adjusted model (Model 2: IRR = 1.12, 95% CI = 0.96, 1.32; *p*-trend = 0.15).

**Figure 2:**
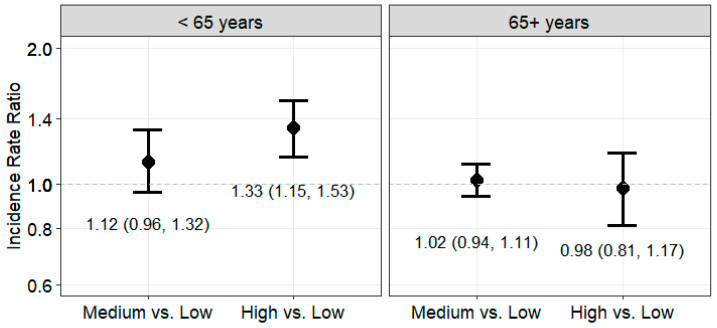
Association between county-level food insecurity tertiles and hepatocellular carcinoma risk stratified by age group at diagnosis and estimated from multi-level Poisson regression models, 21 SEER Registries, 2018–2021. Legend: Estimates are adjusted for sex, race, ethnicity, county-level rates of binge drinking, tobacco smoking, and obesity. County-level food insecurity is modeled as a categorical variable with three levels: low (≤11%), medium (> 1% to <14.5%), and high (≥14.5%). Interaction term *p*-value < 0.001.

As shown in Table 3, the association between county-level FI, modeled as a continuous variable, and HCC risk did not statistically significantly differ by race and ethnicity (Model 2 interaction term *p*-value = 0.69) or by sex (Model 2 interaction term *p*-value = 0.20). Higher county-level FI was associated with higher HCC risk in all racial and ethnic groups except for non-Hispanic Asian or Pacific Islander, as well as in both females and males. There was a statistically significant multiplicative interaction between age at diagnosis and FI, such that a 1-SD increase in county-level FI was associated with a 2% higher HCC risk in the ≥65 age group (Model 2: IRR = 1.02, 95% CI = 1.00, 1.05) and a 15% higher HCC risk in the <65 age group (Model 2: IRR = 1.15, 95% CI = 1.11, 1.19; interaction term *p*-value < 0.001). As shown in Figure 2, being in the highest versus lowest tertile of county-level FI was associated with a 33% higher HCC risk in the <65 age group (Model 2: IRR = 1.33, 95% CI = 1.15, 1.53), but no association was observed in the ≥65 age group (interaction term *p*-value < 0.001).

When stratified by stage at diagnosis, a 1-SD increase in county-level FI was associated with a 7%, 4%, and 16% higher risk of localized, regional, and distant stage HCC, respectively, in the fully adjusted model (Table 4). Being in the highest versus lowest tertile of county-level FI was associated with a 41% higher risk of distant stage HCC in the fully adjusted model (Model 2: IRR = 1.41, 95% CI = 1.22, 1.62). No associations were found for localized or regional stage HCC when FI was analyzed as a categorical variable.

## 4. Discussion

This study provides some of the first data on the association between county-level FI and HCC risk in the U. S. Overall, we observed that higher county-level FI was associated with higher HCC risk after adjusting for patient characteristics (i.e., age at diagnosis, sex, race, and ethnicity) and county-level HCC risk factors (i.e., binge drinking, smoking, and obesity rates). This association was consistently observed in both males and females and across different racial and ethnic groups. However, we found that the association differed by age at diagnosis. Specifically, we found that higher county-level FI was associated with higher HCC risk in the < 65 age group, but we did not find an association in the ≥65+ age group. These findings suggest that county-level FI may be a risk factor for HCC, particularly for early-onset cases. We were unable to determine the reasons for the observed age-specific differences in the association between FI and HCC risk due to the limitations of the data available in our study population. One hypothesis is that these differences may reflect the shift from a higher proportion of viral causes to a higher proportion of non-viral causes, such as MASLD, in younger populations [12,14]. However, further research is needed in clinically well-characterized populations to test this hypothesis. Nevertheless, these findings support the need for enhancing food access through public and private resources, which should be considered as part of prevention and control strategies for this highly fatal disease.

This study provides some of the first data on the relationship between county-level FI and HCC risk. However, previous studies have found that FI is associated with MASLD and fibrosis, which are both established risk factors for HCC [46,47,48,49,50,51]. Although the mechanisms by which FI may increase HCC risk are not fully understood, one key hypothesis involves systemic inflammation. Repeated episodes of food scarcity and hunger may lead to chronic inflammation, promoting central adiposity, insulin resistance [63], and liver damage [64,65]. Additionally, the stress of inadequate food access may elevate cortisol levels, further increasing systemic inflammation [63]. Moreover, dietary changes associated with FI, such as higher consumption of fats, may alter gut microbiota [66], which can contribute to liver inflammation and scarring [66,67,68]. This hypothesis is supported by evidence showing that FI is linked to higher consumption of fats, added sugars, and total calories [69]. Studies have also shown that lower vegetable intake [70,71] and higher saturated fat intake [72] are associated with higher HCC risk. Further, certain dietary patterns, such as the Alternative Healthy Eating Index-2010 (AHEI-2010), have been associated with HCC risk [73,74]. However, a previous ecological study found no association between county-level food environments, defined by the availability of healthy and unhealthy food retailers, and HCC risk [75], suggesting that access to healthy foods alone may not be sufficient to reduce HCC risk. Further research is needed to better understand how FI, diet quality, and the food environment interact to influence HCC risk.

Isolating the association between FI and HCC risk is challenging, especially in ecological studies, because FI is closely intertwined with other social determinants of health that may also contribute to HCC risk. For example, previous research has shown that neighborhood-level poverty is associated with a higher HCC risk, even after adjusting for race, ethnicity, and infections [76]. However, it remains unclear whether this association is attributed to FI or influenced by other HCC-related risk factors, such as infections and alcohol use [77]. Furthermore, poverty not only creates financial barriers to accessing food but also limits access to essential medical resources, such as prescription medications and preventive health care [78,79]. This lack of access is particularly important for people with chronic liver disease, who are at increased risk for HCC [80,81,82]. Individuals with greater financial resources, such as those with comprehensive health insurance, may be more likely to access advanced screening technologies like Magnetic Resonance Imaging with hepato-biliary contrast agents, which improve early detection of small liver tumors and potentially lead to better outcomes [83]. This may be one potential explanation for our finding that FI was associated only with distant-stage disease when stratified by stage at diagnosis, potentially reflecting the impact of FI on access to quality medical care and diagnostic delays. Additionally, underlying conditions associated with HCC risk, such as MASLD, can themselves cause financial strain [84]. As a result, the relationship between FI and HCC risk may be bi-directional, highlighting the need for longitudinal research to better understand this association.

A key limitation of our study is its ecological design, which prevents us from drawing conclusions about the relationship between FI and HCC risk at the individual level. Nevertheless, prior research supports the utility of ecological studies in identifying emerging risk factors for cancers, like HCC, that are increasing over time [85]. Another limitation is the potential for residual confounding, as we could not adjust for individual-level risk factors for HCC, such as alcohol consumption, smoking, and obesity. However, we accounted for these factors at the county-level and individual-level demographic information, including age, sex, race, and ethnicity. We also lacked data on key precursors of HCC, such as viral infections or MASLD; thus, we cannot determine the underlying causes of HCC in our study population. Further research is needed to understand the mediating factors that may explain the observed association between FI and HCC. Strengths of our study include the large sample size of confirmed HCC cases from SEER population-based cancer registries, which represent a substantial portion of the U.S. population. Additionally, we analyzed data from a wide range of counties, capturing diverse levels of FI across the U.S.

## 5. Conclusions

In conclusion, this study supports an association between county-level FI and HCC risk. If confirmed in other studies, these findings support the need for policies and interventions that address FI, particularly in populations at increased risk for HCC. This includes screening for FI in health care settings, such as during routine hepatological clinical care [78]. Screening for FI [86] is essential for identifying at-risk individuals and connecting them to local or federal food assistance programs and other community resources [87]. Community-based initiatives, such as urban gardens [88] and tailored nutrition programs for chronic disease management [89], could also play a key role in supporting and sustaining healthy diets for individuals at increased risk for HCC. Such policies and practices are urgently needed, especially as FI has become a growing public health concern in the U.S. due to the socioeconomic impacts of the COVID-19 pandemic [90,91], which also significantly disrupted HCC treatment and management [92].

## Figures and Tables

**Table 1:** Characteristics of hepatocellular carcinoma cases, 21 SEER Registries, 2018–2021.

Sample Characteristics	n (%)	Mean (SD)
Patient-level characteristics		
Age at diagnosis		
<65 years	17,286 (45)	
≥65 years	21,393 (55)	
Sex		
Female	9115 (24)	
Male	29,564 (76)	
Race and ethnicity		
Hispanic	10,917 (28)	
Non-Hispanic Asian or Pacific Islander	4294 (11)	
Non-Hispanic Black	4870 (12)	
Non-Hispanic White	18,598 (48)	
County-level characteristics		
Food insecurity rate, %		12.9 (3.6)
Binge drinking rate, %		17.6 (2.6)
Tobacco smoking rate, %		19.4 (4.3)
Obesity rate, %		36.0 (4.4)

Abbreviations: SD, standard deviation; SEER, Surveillance, Epidemiology, and End Results.

**Table 2:** Association between county-level food insecurity and hepatocellular carcinoma risk estimated from multi-level Poisson regression models, 21 SEER Registries, 2018–2021.

Measure of County-Level Food Insecurity		Model 1 ^a^	Model 2 ^b^
	Cases	IRR (95% CI)	IRR (95% CI)
Continuous, per 1-SD ^c^	38,679	1.13 (1.08, 1.18)	1.08 (1.06, 1.10)
Tertiles			
Low (≤11.1%)	17,537	ref.	ref.
Medium (>11.1 to <14.5%)	15,504	1.13 (1.00, 1.28)	1.07 (0.96, 1.19)
High (≥14.5%)	5638	1.28 (1.10, 1.49)	1.12 (0.96, 1.32)
*p*-trend		0.01	0.15

Abbreviations: CI, confidence interval; IRR, incidence rate ratio; Q, quartile; SD, standard deviation; SEER, Surveillance, Epidemiology, and End Results. ^a^ Model 1 is adjusted for age at diagnosis, sex, race, and ethnicity. ^b^ Model 2 is adjusted for Model 1 covariates and county-level rates of binge drinking, tobacco smoking, and obesity. ^c^ SD = 3.6%.

**Table 3:** Association between county-level food insecurity, modeled as a continuous variable, and hepatocellular carcinoma risk stratified by patient-level factors and estimated from multi-level Poisson regression models, 21 SEER Registries, 2018-2021.

Stratification Group	Model 1 ^a^		Model 2 ^b^	
		Interaction Term *p*-Value		Interaction Term *p*-Value
	IRR (95% CI) ^c^		IRR (95% CI) ^c^	
Stratified by race and ethnicity		0.51		0.69
Hispanic	1.12 (1.07, 1.18)		1.08 (1.06, 1.11)	
Non-Hispanic API	1.12 (0.97, 1.28)		1.05 (0.87, 1.27)	
Non-Hispanic Black	1.10 (1.03, 1.18)		1.06 (1.00, 1.11)	
Non-Hispanic White	1.14 (1.06, 1.23)		1.09 (1.05, 1.13)	
Stratified by sex		0.18		0.20
Female	1.11 (1.04, 1.17)		1.06 (1.02, 1.10)	
Male	1.14 (1.09, 1.19)		1.09 (1.06, 1.11)	
Stratified by age at diagnosis		<0.001		<0.001
<65 years	1.21 (1.16, 1.25)		1.15 (1.11, 1.19)	
≥65 years	1.07 (1.02, 1.13)		1.02 (1.00, 1.05)	

Abbreviations: API, Asian and Pacific Islander; CI, confidence interval; IRR, incidence rate ratio; SEER, Surveillance, Epidemiology, and End Results. ^a^ Model 1 is adjusted for age at diagnosis, sex, race, and ethnicity. ^b^ Model 2 is adjusted for Model 1 covariates and county-level rates of binge drinking, tobacco smoking, and obesity. ^c^ Effect estimates reflect the association between a 1-standard deviation change in county-level food insecurity and hepatocellular carcinoma risk.

**Table 4:** Association between county-level food insecurity and hepatocellular carcinoma risk stratified by stage at diagnosis and estimated from multi-level Poisson regression models, 21 SEER Registries, 2018–2021.

	Localized (Cases = 19,228)		Regional (Cases = 9990)		Distant (Cases = 6168)	
Measure of County-Level Food Insecurity	Model 1	Model 2	Model 1	Model 2	Model 1	Model 2
	IRR (95% CI)	IRR (95% CI)	IRR (95% CI)	IRR (95% CI)	IRR (95% CI)	IRR (95% CI)
Continuous, per 1-SD ^c^	1.10 (1.03, 1.16)	1.07 (1.02, 1.13)	1.11 (1.08, 1.13)	1.04 (1.01, 1.08)	1.24 (1.16, 1.33)	1.16 (1.11, 1.22)
Tertiles						
Low (≤11.1%)	ref.	ref.	ref.	ref.	ref.	ref.
Medium (>11.1 to <14.5%)	1.07 (0.91, 1.26)	1.03 (0.88, 1.21)	1.11 (1.00, 1.22)	1.03 (0.94, 1.13)	1.26 (1.12, 1.42)	1.17 (1.07, 1.28)
High (≥14.5%)	1.20 (0.98, 1.46)	1.10 (0.90, 1.36)	1.19 (1.04, 1.37)	1.02 (0.86, 1.20)	1.66 (1.39, 1.99)	1.41 (1.22, 1.62)

Abbreviations: CI, confidence interval; IRR, incidence rate ratio; Q, quartile; SD, standard deviation; SEER, Surveillance, Epidemiology, and End Results. ^c^ SD = 3.6%.

## Data Availability

HCC incidence data used in this study is available at https://seer.cancer.gov/data/access.html. Food insecurity data used in this study is available from Feeding America at https://map.feedingamerica.org/. (accessed on 20 August 2023)

## Abbreviations

CFSM, Core Food Security Module; CIs, confidence intervals; FI, Food insecurity (FI); HCC, hepatocellular carcinoma; IRRs, incidence rate ratios (IRRs); MASLD, metabolic dysfunction-associated steatotic disease; NHANES, National Health and Nutrition Examination Survey; SD, standard deviation; SEER, Surveillance, Epidemiology, and End Results.
